# Do community measures impact the effectiveness of a community led HIV testing intervention. Secondary analysis of an HIV self-testing intervention in rural communities in Zimbabwe

**DOI:** 10.1186/s12879-023-08695-x

**Published:** 2023-10-31

**Authors:** Katherine A. Thomas, Euphemia Lindelwe Sibanda, Cheryl Johnson, Constancia Watadzaushe, Getrude Ncube, Karin Hatzold, Mary K. Tumushime, Miriam Mutseta, Nancy Ruhode, Peach P. Indravudh, Frances M. Cowan, Melissa Neuman

**Affiliations:** 1 London School of Hygiene & Tropical Medicine (LSHTM), London, UK; 2 Centre for Sexual Health and HIV/AIDS Research (CeSHHAR) Zimbabwe, Harare, Zimbabwe; 3 Department of International Public Health, Liverpool School of Tropical Medicine, Liverpool, UK; 4 HIV, Hepatitis and STI Department, World Health Organisation, Geneva, Switzerland; 5 Ministry of Health and Child Welfare, Harare, Zimbabwe; 6 Population Services International, Johannesburg, South Africa; 7 Department of Sexual Reproductive Health Rights and Innovations, Population Services International Zimbabwe, Harare, Zimbabwe

**Keywords:** Community cohesion, HIV self-testing, Community-led

## Abstract

**Background:**

There is a growing body of evidence for the role that communities can have in producing beneficial health outcomes. There is also an increasing recognition of the effectiveness and success of community-led interventions to promote public health efforts. This study investigated whether and how community-level measures facilitate a community-led intervention to achieve improved HIV outcomes.

**Methods:**

This is a secondary analysis of survey data from a cluster randomised trial in 40 rural communities in Zimbabwe. The survey was conducted four months after the intervention was initiated. Communities were randomised 1:1 to either paid distribution arm, where HIV self-test (HIVST) kits were distributed by a paid distributor, or community-led whereby members of the community were responsible for organising and conducting the distribution of HIVST kits. We used mixed effects logistic regression to assess the effect of social cohesion, problem solving, and HIV awareness on HIV testing and prevention.

**Results:**

We found no association between community measures and the three HIV outcomes (self-testing, new HIV diagnosis and linkage to VMMC or confirmatory testing). However, the interaction analyses highlighted that in high social cohesion communities, the odds of new HIV diagnosis was greater in the community-led arm than paid distribution arm (OR 2.06 95% CI 1.03–4.19).

**Conclusion:**

We found some evidence that community-led interventions reached more undiagnosed people living with HIV in places with high social cohesion. Additional research should seek to understand whether the effect of social cohesion is persistent across other community interventions and outcomes.

**Trial registration:**

PACTR201607001701788.

**Supplementary Information:**

The online version contains supplementary material available at 10.1186/s12879-023-08695-x.

## Background

There is an increasing wealth of research highlighting the positive effect of community engagement on public health efforts [1].Engaging community members through a community led intervention offers a more sustainable, ethical and financially feasible option to programs driven by external organisations [2]. A community-led intervention involves empowering and supporting community members to gain the skills and knowledge to be able to identify the issues causing poor health and how to implement programmes to tackle these problems effectively [3, 4]. The success of a community-led model relies on the community members and leaders accepting that health is promoted at the level of community, rather than solely at the individual level [5]. Research has noted the beneficial role of community participation in improving health outcomes [6], including HIV [7, 8].Related to this, is the concept of social cohesion which has been defined as “the degree to which an individual finds trust, solidarity, connectedness, and sense of belonging within a group in society” [9]. Social cohesion has been found to be associated with better HIV outcomes, in particular promoting HIV testing [9–11].

Community-led distribution of HIV self-testing has been shown to be a particularly effective approach for increasing access to testing and onward treatment [12] [13]. As a self-directed approach, HIVST enables individuals to collect their own specimen, either blood or oral fluid, conduct the test, and interpret the results [14]. Research suggests that HIVST has high acceptability amongst low- and middle-income countries [15] and has the potential to increase HIV testing in populations that typically have lower testing uptake [16]. HIVST is therefore seen as a strategy that can tackle barriers to testing, and result in increased awareness and use of prevention and treatment services.

Previous literature on the effect of social cohesion on HIV outcomes has not always been consistent, this paper seeks to understand the relationship focusing on a community level measure of cohesion. In addition, there has been minimal literature on the role of community cohesion on the effectiveness of interventions delivered at the community level. Using data from a cluster randomised trial this paper seeks to address both the effect of community measures on HIV outcomes, and whether this association is stronger in the community-led arm compared to the community-based arm.

## Methodology

### Study design

The study was conducted as part of the Unitaid STAR (Self-Testing Africa) Initiative, which aims to answer key public health questions about HIVST and to increase effective use of HIVST, including ensuring adequate linkage to treatment and prevention services [17]. This is a secondary analysis of cross-sectional survey data from a cluster randomised trial which compared HIV outcomes between community-led and paid distribution of HIVST in rural Zimbabwe communities [18].

### Setting

The trial was conducted in 40 rural communities across Zimbabwe. Communities were defined as headman units, an official administrative unit through which rural community-level activities are typically implemented in Zimbabwe. Communities were only eligible if they contained at least three census enumeration areas (EAs) and were separated by at least 20km. Communities were randomly selected. Communities were randomised 1:1 to either a paid distribution arm, whereby HIVST kits were distributed by a paid distributor, or community-led whereby members of the community were responsible for organising and conducting the distribution of HIVST kits. Within the community-led arm, community engagement activities were carried out over a period of 2–3 weeks, both to introduce the idea of community-led HIVST and to teach communities the concept of undetectable = untransmissible (U = U). Paid HIVST distributors in each community were given training to promote and support HIVST and to promote uptake of confirmatory testing for those with reactive tests and HIV prevention (VMMC) following a negative self-test. Paid HIVST distributors completed door to door visits to distribute kits. Both community led and community based implementors received training materials on HIV, HIVST and treatment for preventing transmission. In addition to this, the community-led arm also received posters and flyers which could be distributed to the community. Observed community-led distribution models included 1) door-to-door distribution only or 2) door-to-door distribution as well as collection of kits directly from distributors at their homes or at various locations in the communities [19].

### Participants

In order to be eligible to complete the survey and be included in this analysis, individuals had to be over the age of 16 and have lived in the community throughout the distribution of HIVST. Within each cluster, three enumeration areas (EAs) were selected, within each enumeration area one in two households were randomly selected to participate.. Those included in the analysis are those that were eligible, from selected households, and who gave consent to participate in the follow up survey.

### Data collection

Responses from the representative population-based survey were entered via Audio Computer-Assisted Self-Interviewing (ACASI) devices to ensure participants were comfortable and could answer honestly. The survey was administered in the local language and included questions about both a household questionnaire including assets, household hunger, and an individual questionnaire including demographic characteristics, sexual behaviour, HIV testing and usage of HIV services. The survey also included a six-item measure of community cohesion (*reliability coefficient: 0.8804*) which was validated by Lippman et al. in high HIV prevalence settings in South Africa [20]. A ten-item measure of community HIV awareness (*reliability coefficient: 0.9174)* and an eleven-item measure of community problem solving (*reliability coefficient: 0.9472)* were created. Both HIV awareness and community problem solving have not been validated. These three variables were then divided into terciles to create low, medium and high categories. The community measures are shown in Table 1. Dried blood spot (DBS) samples were also collected to test for HIV, viral load and current use of ART. The household food insecurity was assessed according to the Household Food Insecurity Access Scale. Both assets & food insecurity were generated using principal components analysis.

**Table 1:** Exposure variables

***Community Cohesion***
1. People in this village are willing to help their neighbours
2. This is a close-knit community
3. People in this village can be trusted
4. People in this village generally get along well with each other
5. People in this village share the same values
6. People in this village look out for each other
***Community HIV Awareness***
1. People in your village are concerned about HIV
2. People in your village consider HIV/AIDS an important issue
3. People in your village talk openly about HIV
4. People in your village believe that HIV impacts the community
5. People in your village talk about HIV/AIDS at community meetings
6. People in your village work together to prevent HIV from spreading
7. People in your village work together to reduce the effects of HIV
8. People in your village believe they can change the course of the HIV/AIDS epidemic
9. People in your village exchange information about HIV/AIDS
10. People in your village take HIV/AIDS seriously
***Community problem solving***
1. People work together to solve problems in the village
2. People in your village talk to each other about how to solve village problems
3. People in your village enjoy discussing different ways to solve village problems
4. People in your village are open to hearing different views about community problems and solutions
5. People in your village volunteer to help solve village problems
6. People in your village think about why there are problem
7. People in your village think about why there are problems so they can address the course of problems
8. There is a lot of cooperation between groups in the village
9. People in this village not only talk about problems but they also try to solve them
10. If your community fails to resolve a community problem they will learn from that experience and do a better job when they try to solve the problem in the future
11. If leaders in the village fails to resolve a village problem the villagers will work together to find a solution

### Outcomes

The following outcomes of this study were collected four months after the intervention occurred:

1. Self-testing uptake: Based on survey responses, those using an HIVST and not reporting ART use during the study period, out of the whole survey population.
2. New HIV diagnosis: Based on dried blood spot samples collected at the time of the survey.
3. Self-report linkage: Based on the survey responses, those receiving a reactive HIVST result and individual reported confirmatory testing or individual was circumcised after testing negative for HIV.

New HIV diagnosIs was measured using a dried blood spot to minimise bias, particularly due to the sensitivity of people reporting their HIV status.

### Statistical analyses

Individual level cohesion, HIV awareness and problem-solving scores were calculated using the average item response ranging from 1 (strongly disagree) to 5 (strongly agree). From these scores, community level cohesion, HIV awareness and problem solving were calculated as the median score of individuals within the cluster, the variable was then categorised by terciles (low, medium, high). Community level variables were used for the analysis. A logistic regression with random effects to account for clustering at the level of the community was fitted to assess how the outcomes differed by the three community measures and HIVST distribution method. All models are adjusted for several confounding factors: age, sex, ethnicity, religion, salary, marital status, education, household hunger and assets. Analysis was carried out using Stata 16.1 [21].

## Results

### Study population

Out of the study population of 11,150, over half (55%) were female participants. The mean age of participants was 36 years old, with the youngest participant aged 16. The majority of the participants (80%) were of Shona ethnicity and most of the participants had at least some level of education (93%).

The proportion of people of Ndebele ethnicity reduced with increasing social cohesion (see Additional file 1). Contrastingly, the proportion of people of Ndebele ethnicity increased considerably from low HIV awareness (3%) to high HIV awareness communities (26%) (Additional file 2). HIV awareness was also associated with ethnicity, education, food insecurity and assets. Community problem solving was associated with ethnicity, education and food insecurity (Additional file 3). The proportion of study participants that had severe food insecurity was lowest at 17% in low problem solving communities and increased with increasing problem solving to 25% in high problem solving communities.

Out of a total of 11,150 participants 2,737 (24.5%) had an HIV self-test and 408 (3.6%) had a new HIV diagnosis. Of the 2,737 participants who used a self-test and were not using ART, 278 (10.2%) linked to either confirmatory testing or VMMC.

### Effect of community measures on HIV outcomes

Community social cohesion had no effect on all three HIV outcomes (Table 2).

**Table 2:** Adjusted odds ratios for effect of community social cohesion on HIV outcomes

Cohesion					
	Self-test coverage (*n* = 11,150)				*P*-value*
	Didn’t take self-test	Took up self-test	Crude OR (95% CI)	Adjusted^a^ OR (95% CI)	
Low	1975 (74.7)	669 (25.3)	1		
Medium	3263 (77.8)	929 (22.2)	0.85 (0.53–1.35)	0.82 (0.52–1.20)	0.57
High	3175 (73.6)	1139 (26.4)	1.03 (0.64–1.66)	1.02 (0.63–1.65)	
	New HIV Diagnosis (*n* = 11,150)				
	Not new diagnosis	New Diagnosis	Crude OR (95% CI)	Adjusted^a^ OR (95% CI)	
Low	2580 (97.6)	64 (2.4)	1		
Medium	4082 (97.4)	110 (2.6)	1.02 (0.58–1.78)	1.08 (0.63–1.87)	0.85
High	4222 (97.9)	92 (2.1)	0.84 (0.47–1.50)	0.94 (0.53–1.65)	
	Linkage (*n* = 2737)				
	Didn't link	Linked	Crude OR (95% CI)	Adjusted^a^ OR (95% CI)	
Low	605 (90.4)	64 (9.6)	1		0.89
Medium	815 (87.7)	114 (12.3)	1.18 (0.75–1.87)	1.12 (0.64–1.95)	
High	1039 (91.2)	100 (8.8)	0.92 (0.58–1.46)	1.00 (0.57–1.76)	

^*^p-value from likelihood ratio test

^a^adjusted for age, sex, salary, education, marital status, household hunger and assets

Table 3 highlights that community HIV awareness was not associated with any of the three outcome variables.

**Table 3:** Adjusted odds ratios for effect of community HIV awareness on HIV outcomes

Community HIV Awareness					
	Self-test coverage (*n* = 11,150)				*P*-value*
	Didn’t take self-test (%)	Took up self-test (%)	Crude OR (95% CI)	Adjusted^a^ OR (95% CI)	
Low	2935 (71.1)	1194 (28.9)	1		0.10
Medium	2573 (75.6)	831 (24.4)	0.92 (0.60–1.40)	0.92 (0.59–1.41)	
High	2905 (80.3)	712 (19.7)	0.62 (0.41–0.94)	0.63 (0.41–0.98)	
	New HIV Diagnosis (*n* = 11,150)				
	Not new diagnosis	New Diagnosis	Crude OR (95% CI)	Adjusted^a^ OR (95% CI)	
Low	4040 (97.8)	89 (2.2)	1		0.29
Medium	3304 (97.1)	100 (2.9)	1.45 (0.86–2.47)	1.38 (0.83–2.29)	
High	3540 (97.9)	77 (2.1)	0.98 (0.57–1.67)	0.94 (0.55–1.59)	
	Linkage (*n* = 2737)				
	Didn't link	Linked	Crude OR (95% CI)	Adjusted^a^ OR (95% CI)	
Low	1075 (90.0)	119 (10.0)	1		0.92
Medium	745 (89.7)	86 (10.30)	1.1 (0.70–1.72)	1.09 (0.65–1.86)	
High	639 (89.8)	73 (10.20)	1.1 (0.70–1.72)	1.10 (0.63–1.93)	

^*^p-value from likelihood ratio test

^a^adjusted for age, sex, salary, education, marital status, household hunger and assets

Similarly to HIV awareness, there was no evidence of an association between community problem solving and the three outcome variables (Table 4).

**Table 4:** Adjusted odds ratios for effect of community problem solving on HIV outcomes

Community problem solving						
	Self-test coverage (*n* = 11,150)					*P*-value*
	Didn’t take self-test	Took up self-test		Crude OR (95% CI)	Adjusted* OR (95% CI)	
Low	2890 (71.4)	1158 (28.6)		1		0.17
Medium	2512 (76.6)	767 (23.4)		0.76 (0.49–1.18)	0.73 (0.47–1.13)	
High	3011 (78.8)	812 (21.2)		0.66 (0.43–1.01)	0.66 (0.43–1.02)	
	New HIV Diagnosis (*n* = 11,150)					
	Not new diagnosis	New Diagnosis		Crude OR (95% CI)	Adjusted^a^ OR (95% CI)	
Low	3967 (98.0)	81 (2.0)		1		0.47
Medium	3195 (97.4)	84 (2.6)		1.27 (0.73–2.21)	1.32 (0.77–2.25)	
High	3722 (97.4)	101 (2.6)		1.37 (0.80–2.34)	1.35 (0.80–2.30)	
	Linkage (*n* = 2737)					
	Didn't link		Linked	Crude OR (95% CI)	Adjusted^a^ OR (95% CI)	
Low	1058 (91.4)	100 (8.6)		1		0.81
Medium	676 (88.1)	91 (11.9)		1.21 (0.77–1.90)	0.92 (0.53–1.59)	
High	725 (89.3)	87 (10.7)		1.24 (0.81–1.92)	1.11 (0.64–1.91)	

^*^p-value from likelihood ratio test

^a^adjusted for age, sex, salary, education, marital status, household hunger and assets

### Trial effectiveness of community-led interventions in communities with stronger community measures

As shown in Table 5, in communities with high social cohesion, the odds of a new HIV diagnosis were over double (OR 2.08 95% CI 1.03–4.19 *p*-value 0.04) in community-led compared to the paid distribution arm. There was no evidence of interaction between social cohesion and community arm for self-testing or linkage.

**Table 5:** Estimates of the association between allocation arm and HIV outcomes stratified by community social cohesion

**Self-testing**	Allocation Arm	Self-test uptake (%)	S–S adjusted OR (95% CI)	*P*-value for interaction
Low	Paid distribution	214 (24.0)	1	
	Community-led	455 (26.0)	1.23 (0.62–2.4)	
Medium	PD	418 (23.4)	1	0.12
	Community-led	511 (21.2)	0.75 (0.44–1.28)	
High	PD	876 (31.4)	1	
	Community-led	263 (17.3)	0.47 (0.26–0.84)	
**New HIV diagnosis**	Allocation Arm	HIV Diagnosis (%)	S–S adjusted OR (95% CI)	*P*-value for interaction
Low	Paid distribution	26 (2.9)	1	
	Community-led	38 (2.2)	0.81 (0.36–1.77)	
Medium	Paid distribution	57 (3.2)	1	0.04
	Community-led	53 (2.2)	0.61 (0.32–1.16)	
High	Paid distribution	45 (1.6)	1	
	Community-led	47 (3.1)	2.08 (1.03–4.19)	
**Linkage**	Allocation Arm	Linkage (%)	S–S adjusted OR (95% CI)	*P*-value for interaction
Low	Paid distribution	15 (7.0)	1	
	Community-led	49 (10.8)	1.64 (0.78–3.45)	
Medium	Paid distribution	40 (9.6)	1	0.89
	Community-led	74 (14.5)	1.32 (0.76–2.30)	
High	Paid distribution	70 (8.0)	1	
	Community-led	30 (11.4)	1.50 (0.83–2.71)	

There was evidence of possible interaction between community HIV awareness and trial effectiveness (Table 6). In low HIV awareness communities, the community led arm had a lower odds of self-testing uptake (OR 0.41 95% CI 0.24–0.69 *p*-value 0.03). However, in contrast, in low HIV awareness communities, the community led arm resulted in 2.6 increased odds of linkage (95% CI 1.58–4.28 *p*-value 0.03) compared to paid distribution clusters.

**Table 6:** Estimates of the association between allocation arm and HIV outcomes stratified by community HIV awareness

**Self-testing**	Allocation Arm	Self-test uptake (%)	S–S adjusted OR (95% CI)	*P*-value for interaction
Low	Paid distribution	799 (36.8)	1	
	Community-led	395 (20.2)	0.41 (0.24–0.69)	
Medium	Paid distribution	449 (22.7)	1	0.03
	Community-led	382 (26.8)	1.06 (0.62–1.81)	
High	Paid distribution	260 (19.7)	1	
	Community-led	452 (19.7)	0.98 (0.58–1.66)	
**New HIV diagnosis**	Allocation Arm	HIV Diagnosis (%)	S–S adjusted OR (95% CI)	*P*-value for interaction
Low	Paid distribution	35 (1.6)	1	
	Community-led	54 (2.7)	2.00 (0.96–4.19)	
Medium	Paid distribution	58 (2.9)	1	0.09
	Community-led	42 (3.0)	0.88 (0.43–1.79)	
High	Paid distribution	35 (2.7)	1	
	Community-led	42 (1.8)	0.67 (0.32–1.39)	
**Linkage**	Allocation Arm	Linkage (%)	S–S adjusted OR (95% CI)	*P*-value for interaction
Low	Paid distribution	54 (6.8)	1	
	Community-led	65 (16.5)	2.60 (1.58–4.28)	
Medium	Paid distribution	46 (10.2)	1	0.03
	Community-led	40 (10.5)	0.98 (0.57–1.68)	
High	Paid distribution	25 (9.6)	1	
	Community-led	48 (10.6)	1.13 (0.62–2.03)	

Similarly, to self-testing in communities with low HIV awareness, communities with low problem solving (Table 7) had a lower odds of self-testing uptake in the community-led arm (0.48 95% CI 0.28–0.84 *p*-value for interaction 0.06). Within medium problem-solving communities, the odds of linkage were over double that within the community led arm (2.02 95% CI 1.10–3.72 *p*-value 0.39).

**Table 7:** Estimates of the association between allocation arm and HIV outcomes stratified by community problem solving

**Self-testing**	Allocation Arm	Self-test uptake (%)	S–S adjusted OR (95% CI)	*P*-value for interaction
Low	Paid distribution	807 (35.1)	1	
	Community-led	351 (20.1)	0.48 (0.28–0.84)	
Medium	Paid distribution	360 (24.9)	1	0.06
	Community-led	407 (22.2)	0.67 (0.38–1.18)	
High	Paid distribution	341 (19.8)	1	
	Community-led	471 (22.4)	1.17 (0.68–2.02)	
**New HIV diagnosis**		HIV Diagnosis (%)	S–S adjusted OR (95% CI)	*P*-value for interaction
Low	Paid distribution	35 (1.5)	1	
	Community-led	46 (2.6)	1.69 (0.81–3.55)	
Medium	Paid distribution	39 (2.7)	1	0.22
	Community-led	45 (2.5)	0.96 (0.46–2.02)	
High	Paid distribution	54 (3.1)	1	
	Community-led	47 (2.2)	0.68 (0.34–1.35)	
**Linkage**		Linkage (%)	S–S adjusted OR (95% CI)	*P*-value for interaction
Low	Paid distribution	64 (7.9)	1	
	Community-led	36 (10.3)	1.35 (0.79–2.31)	
Medium	Paid distribution	26 (7.2)	1	0.39
	Community-led	65 (16.0)	2.02 (1.10–3.72)	
High	Paid distribution	35 (10.2)	1	
	Community-led	52 (11.0)	1.12 (0.64–1.94)	

## Discussion

These results indicate that a community-led intervention may be more effective in high social cohesion communities and high problem solving communities compared to the paid distributor arm.

In high social cohesion communities, the odds of new HIV diagnosis were over double in clusters with the community-led intervention (OR 2.08 95% CI 1.03–4.19). Notably, the proportion of new HIV diagnoses was considerably higher for the community-led intervention arm than the paid distribution intervention arm within high social cohesion communities, despite the fact that self-testing was not, which may indicate better targeting of self-tests to people who need to test [13]. As supported by other studies, this suggests that the community-led intervention maybe more effective at reaching those who have been undiagnosed [22]. Due to social connectedness, increased trust and care for one another, effective dissemination of U = U messaging may have facilitated reaching those who were undiagnosed in high social cohesion communities. These results suggest communities that are closely knit are more likely to know who is at high risk and needs to be provided with a test, suggesting interventions are more successful when focusing on targeting high risk populations that are within the community.

Unlike the paid distribution arm, the community led model also allowed for testing in other locations as well as door-to-door, those at higher risk of HIV and more likely to test positive would be more likely to be concerned about privacy and thus utilise the opportunities to test outside of the house. In addition, the community-led intervention may have fostered a sense of ownership, creating a more secure and enabling environment to test, compared to the paid distributor arm where the HIVST program was implemented by a paid distributor.

This is reflected in the results which show that those in the community-led arm had over double (OR 2.08 95% CI 1.03–4.19) the odds of HIV self-testing compared to community-based arm in high social cohesion communities. This is aligned with previous research that has found higher social cohesion to increase HIV testing [10, 23]. Literature has suggested that increased cohesion promotes trust and community empowerment which results in behaviour change.

Within low HIV awareness communities, clusters allocated to the community-led model had 0.41 (95% CI 0.24–0.69 *p*-value 0.03) the odds of self-testing compared to those allocated to the paid distribution arm. These results may reflect the challenge of communities that have low HIV awareness to drive their own HIV programme compared to those where a paid facilitator is promoting self testing uptake. HIV awareness has previously been found to be related to stigma towards people living with HIV [24], therefore those with low HIV awareness could have higher levels of HIV stigma making participation and engagement with self testing more challenging. Also, within low HIV awareness communities, the community led intervention was associated with a 2.6 (95% CI 1.58–4.28 *p*-value 0.03) increased odds of self reported linkage compared to paid distribution clusters. This increase in odds was driven primarily by two clusters who had a higher proportion of linkage compared to other clusters. The high rate of linkage in these communities can be attributed to long standing health workers that had a strong relationship with the community (N Ruhode, personal communication, 26 May). This is supported by research noting that trust within a community typically promotes healthier behaviours [22].

Our results also indicated evidence of interaction between community problem solving and trial effectiveness. The results highlighted a dose response for self-testing, with the odds of reported self-testing increasing from low problem-solving communities (community-led OR 0.48 95% CI 0.28–0.84) to high community problem solving communities (community-led OR 1.17 95% CI 0.68–2.02). These results could be driven by some communities within this group that are better at problem solving being more proactive in running the community-led intervention, resulting in an increase in self testing.

There is a growing body of evidence for the role of social cohesion and community ownership in promoting positive health outcomes [1, 12, 25]. Whilst community problem solving could be attributed to communities working towards positive goals, cohesive communities do not necessarily translate into positive change. Strong social ties that bond a community together also have the ability to exclude people outside the group [26]. Therefore, the valence of social cohesion needs to be considered in the specific context of each community [27], exploring differences both within and between communities.

The results of this study need to be considered in context of its limitations. Due to the nature of the study design, temporality could have caused bias. Whilst the survey aimed to gather precisely when participants most recent HIVSTs were conducted, the date of the HIVST could have been recalled incorrectly. Therefore, it is difficult to be certain that the intervention occurred before participants had a new HIV diagnosis or linked to preventative care. Also, it is hard to disentangle whether the community measures were pre-existing and improved the effectiveness of the intervention or whether the intervention improved the community measures, particularly social cohesion. However, due to the short period of the intervention it is unlikely to have been able to improve community measures such as social cohesion enough to have influenced the intervention.

Due to the number of statistical comparisons in the analyses of these results, it is possible some significant associations may have been obtained by chance. This paper describes an exploratory analyses of the data and therefore results have been interpreted with caution and indicate emerging patterns or areas of future research focus, as opposed to drawing conclusions.

Despite the limitations, this study contributes to existing literature on the role of community-led interventions on HIV outcomes. Our results highlight that community-led interventions have the ability to reach high risk populations in communities that have stronger existing community measures. This study uses community measures validated for research purposes and also assesses these at the community-level as opposed to the individual level. This enables our findings to be more meaningful in understanding how the context of a community interacts with health outcomes.

## Conclusion

This study indicates that community-led interventions could potentially have a greater effect in communities with stronger social cohesion and community problem solving [28, 29]. In communities that are cohesive, HIVST programmes should seek to involve community members in the planning and implementation stages to be more effective in reaching community members and improving HIV outcomes. Implementation of community-led interventions should be conducted on the basis that knowledge of a community gives any intervention the opportunity to strengthen capacity and improve local resources [30]. Community-led interventions need to incorporate and consider the wider social dynamics of a community to ensure the success of a community-led intervention.

### Supplementary Information

**Additional file 1.** Distribution of surveyed population by community cohesion.

**Additional file 2.** Distribution of surveyed population by community HIV awareness.

**Additional file 3.** Distribution of surveyed population by community problem solving.

## Data Availability

Data are available upon request. De-identified data are available from Melissa Neuman (Melissa.Neuman@lshtm.ac.uk) upon request.

## Abbreviations

ART: Antiretroviral therapy
CI: Confidence intervals
WHO: World Health Organization
HIV: Human Immunodeficiency Viruses
PLWHIV: People Living With HIV
UNAIDS: The Joint United Nations Programme on HIV and AIDS
HIVST: HIV Self Testing
U = U: Undetectable = Untransmissable
STI: Sexually Transmitted Infection
RCT: Randomised Controlled Trial
VMMC: Voluntary Male Circumcision
EA: Enumeration Areas
EA: Enumeration Area
PCA: Principal Components Analysis
MSM: Men Who Have Sex With Men
AIDS: Acquired Immune Deficiency Syndrome
UN: United Nations
