# Supplementary material for: Do community measures impact the effectiveness of a community led HIV testing intervention. Secondary analysis of an HIV self-testing intervention in rural communities in Zimbabwe
Source: BMC Infect Dis. 2023 Oct 31;22(Suppl 1):974. doi: 10.1186/s12879-023-08695-x (PMC10617038; doi:10.1186/s12879-023-08695-x)
Supplement: Supplementary file 1 — Additional file 1. Distribution of surveyed population by community cohesion. [file 12879_2023_8695_MOESM1_ESM.pdf]

Additional file 1: Distribution of surveyed population by community cohesion				
**Variable**	**Social Cohesion Measure**			
	Low	Medium	High	p-value
**Allocation**				
CBD	892	1,784	2,791	0.001
CLD	1,752	2,408	1,523	
**Age (missing=8)**				
16-19 years	413 (15.6)	712 (17.0)	698 (16.2)	
20-25 years	441 (16.7)	754 (18.0)	762 (17.7)	0.001
26-35 years	636 (24.1)	1,004 (24.0)	944 (21.9)	
36-50 years	729 (27.6)	1,027 (24.5)	1,073 (24.9)	
50+ years	425 (16.1)	691 (16.5)	833 (19.3)	
**Sex**				
Male	1,170 (44.3)	1,916 (45.7)	1,984 (46.0)	0.342
Female	1,474 (55.8)	2,276 (54.3)	2,330 (54.0)	
**Ethnicity (missing=32)**				
Shona	1,920 (72.6)	3,194 (76.2)	3,845 (89.1)	0.001
Ndebele	406 (15.4)	576 (13.8)	233 (5.4)	
Other	312 (11.8)	407 (9.7)	222 (5.2)	
**Religion**				
Apostolic	976 (36.9)	1,638 (39.1)	1,647 (38.2)	0.201
Non-Apostolic	1,668 (63.1)	2,554 (60.9)	2,667 (61.8)	
**Salary**				
No	1,958 (74.1)	3,183 (75.9)	3,302 (76.5)	0.035
Yes	663 (25.1)	956 (22.8)	957 (22.2)	
**Marital Status (missing=155)**				
Married	1,605 (60.7)	2,558 (61.0)	2,598 (60.2)	0.620
Never married	602 (22.8)	994 (23.7)	1,005 (23.3)	
Widowed/separated	404 (15.3)	580 (13.8)	649 (15.0)	
**Education**				
None	219 (8.3)	313 (7.5)	258 (6.0)	
Some primary	873 (33.0)	1,437 (34.3)	1,276 (29.6)	0.001
Some secondary	587 (22.2)	1,119 (26.7)	1,102 (25.5)	
Qualifications	965 (36.5)	1,323 (31.6)	1,678 (38.9)	
**Food Insecurity**				
Little	977 (38.6)	1,786 (44.3)	2,177 (52.7)	0.001
Moderate	887 (35.1)	1,455 (36.1)	1,310 (31.7)	
Severe	666 (26.3)	794 (19.7)	641 (15.5)	
	**Assets (missing= 5,391)**			
Lowest	637 (25.4)	679 (17.0)	593 (14.6)	

Second	444 (17.7)	840 (21.0)	751 (18.4)	
Middle	402 (16.0)	794 (19.9)	909 (22.3)	0.001
Fourth	432 (17.2)	814 (20.4)	927 (22.7)	
Highest	592 (23.6)	868 (21.7)	897 (22.0)	
