# Supplementary material for: Do community measures impact the effectiveness of a community led HIV testing intervention. Secondary analysis of an HIV self-testing intervention in rural communities in Zimbabwe
Source: BMC Infect Dis. 2023 Oct 31;22(Suppl 1):974. doi: 10.1186/s12879-023-08695-x (PMC10617038; doi:10.1186/s12879-023-08695-x)
Supplement: Supplementary file 2 — Additional file 2. Distribution of surveyed population by community HIV awareness. [file 12879_2023_8695_MOESM2_ESM.pdf]

Additional file 2: Distribution of surveyed population by community HIV awareness				
**Variable**	**HIV Awareness Measure**			
	Low	Medium	High	p-value
**Allocation**				
CBD	2,170 (52.6)	1,979 (58.1)	1,318 (36.4)	0.001
CLD	1,959 (47.4)	1425 (41.9)	2,299 (63.6)	
**Age (missing=8)**				
16-19 years	652 (15.8)	591 (17.4)	580 (16.0)	
20-25 years	697 (16.9)	644 (18.9)	616 (17.0)	
26-35 years	896 (21.7)	769 (22.6)	919 (25.4)	0.001
36-50 years	1,081 (26.2)	813 (23.9)	935 (25.9)	
50+ years	799 (19.4)	584 (17.2)	566 (15.7)	
**Sex**				
Male	1,879 (45.5)	1,558 (45.8)	1,633 (45.2)	0.871
Female	2,250 (54.5)	1,846 (54.2)	1,984 (54.9)	
**Ethnicity (missing=32)**				
Shona	3,755 (90.9)	2,892 (87.6)	2,222 (61.4)	
Ndebele	103 (2.5)	167 (4.9)	948 (26.2)	0.001
Other	255 (6.2)	247 (7.3)	439 (12.1)	
**Religion**				
Apostolic	1,667 (40.4)	1,245 (36.6)	1,349 (37.3)	0.001
Non-Apostolic	2,462 (59.6)	2,159 (63.4)	2,268 (62.7)	
**Salary**				
No	3,191 (77.3)	2,580 (75.8)	2,672 (73.9)	
Yes	886 (21.5)	784 (23.0)	906 (25.0)	0.006
**Marital Status (missing=155)**				
Married	2,558 (62.0)	2,004 (58.9)	2,199 (60.8)	
Never married	906 (21.9)	833 (24.5)	862 (23.8)	0.048
Widowed/separated	602 (14.6)	526 (15.5)	505 (14.0)	
**Education**				
None	270 (6.5)	230 (6.8)	290 (8.0)	
Some primary	1,324 (32.1)	994 (29.2)	1,268 (35.1)	0.001
Some secondary	992 (24.0)	903 (26.5)	913 (25.2)	
Qualifications	1,543 (37.4)	1,277 (37.5)	1,146 (31.7)	
**Food Insecurity**				
Little	2,033 (51.3)	1,473 (45.1)	1,434 (41.4)	0.001
Moderate	1,268 (32.0)	1,126 (34.5)	1,258 (36.3)	
Severe	663 (16.7)	665 (20.4)	773 (22.3)	
**Assets (missing= 5,391)**				
Lowest	558 (14.3)	582 (18.1)	769 (22.4)	

Second	733 (18.7)	687 (21.3)	615 (17.9)	
Middle	877 (22.4)	628 (19.5)	600 (17.5)	0.001
Fourth	891 (22.8)	640 (19.8)	642 (18.7)	
Highest	858 (21.90)	688 (21.3)	811 (23.6)	
