# Supplementary material for: Do community measures impact the effectiveness of a community led HIV testing intervention. Secondary analysis of an HIV self-testing intervention in rural communities in Zimbabwe
Source: BMC Infect Dis. 2023 Oct 31;22(Suppl 1):974. doi: 10.1186/s12879-023-08695-x (PMC10617038; doi:10.1186/s12879-023-08695-x)
Supplement: Supplementary file 3 — Additional file 3. Distribution of surveyed population by community problem solving. [file 12879_2023_8695_MOESM3_ESM.pdf]

Additional file 3: Distribution of surveyed population by community problem solving				
**Variable**	**Problem Solving Measure**			
	Low	Medium	High	p-value
**Allocation**				
CBD	2,302 (56.9)	1,445 (44.1)	1,720 (45.0)	
CLD	1,746 (43.1)	1,834 (55.9)	2,103 (55.0)	
**Age (missing=8)**				
16-19 years	691 (17.1)	500 (15.3)	632 (16.5)	
20-25 years	642 (15.9)	652 (19.9)	663 (17.3)	0.001
26-35 years	821 (20.3)	897 (27.4)	866 (22.7)	
36-50 years	1,029 (25.4)	810 (24.7)	990 (25.9)	
50+ years	861 (21.3)	417 (12.7)	671 (17.6)	
**Sex**				
Male	1,732 (42.8)	1,647 (50.2)	1,691 (44.2)	0.001
Female	2,316 (57.2)	1,632 (49.8)	2,132 (55.8)	
**Ethnicity (missing=32)**				
Shona	3,630 (89.7)	2,929 (89.3)	2,400 (62.8)	
Ndebele	205 (5.1)	56 (1.7)	957 (25.0)	0.001
Other	199 (4.9)	286 (8.7)	456 (11.9)	
**Religion**				
Apostolic	1595 (39.4)	1,359 (41.5)	1,307 (34.2)	0.001
Non-Apostolic	2,453 (60.6)	1,920 (58.6)	2,516 (65.8)	
**Salary**				
No	3,159 (78.0)	2,444 (74.5)	2,840 (74.3)	0.001
Yes	844 (20.9)	787 (24.0)	945 (24.7)	
**Marital Status (missing=155)**				
Married	2,421 (59.8)	2,074 (63.3)	2,266 (59.3)	
Never married	919 (22.7)	746 (22.8)	936 (24.5)	0.001
Widowed/separated	655 (16.2)	408 (12.4)	570 (14.9)	
**Education**				
None	253 (6.3)	224 (6.8)	313 (8.2)	
Some primary	1,204 (29.7)	1,105 (33.7)	1,277 (33.4)	0.001
Some secondary	983 (24.3)	878 (26.8)	947 (24.8)	
Qualifications	1,608 (39.7)	1,072 (32.7)	1,286 (33.6)	
**Food Insecurity**				
Little	1,986 (51.1)	1,551 (49.5)	1,403 (38.2)	0.001
Moderate	1,245 (32.1)	1,040 (33.2)	1,367 (37.2)	
Severe	654 (16.8)	543 (17.3)	904 (24.6)	
